# Supplementary material for: School Fruit and Vegetables Scheme: Characteristics of Its Implementation in the European Union from 2009/10 to 2016/17
Source: Nutrients. 2022 Jul 26;14(15):3069. doi: 10.3390/nu14153069 (PMC9370373; doi:10.3390/nu14153069)
Supplement: Supplementary file 1 [file nutrients-14-03069-s001.zip › nutrients-1804297-Document S1.pdf]

ANNEX IV: SUMMARY REPORT 'STRATEGY' 2014-2015

SUMMARY REPORT

**1 Member State**		SPAIN	**Region** (if applicable)	
**2 Submission date**		30-04-2014	**Commission Reception date**	
**3 National scheme**		YES		
**4 Regional scheme**			Convergence region (relevant NUTS level)	
**5 Single contact point**	Name	MARIA JOSEFA LUESO SORDO		
	Institution	MINISTERIO DE AGRICULTURA, ALIMENTACION Y MEDIO AMBIENTE Subdirección General de Promoción Alimentaria		
	Mailing Address	Pº INFANTA ISABEL 1 28071 MADRID ESPAÑA		
	Tel	0034913475120		
	Fax	0034913475120		
	E-mail	planfrutaescuelas@magrama.es y sgpromocion@magrama.es		

6 Budget (in €) (indicative)

	Year 2010/2011	Year 2011/2012	Year 2012/2013	Year 2013/2014	Year 2014/2015
**Total BUDGET** (EU + MS)					14.218.825
**EU funding**					10.724.670
Regional schemes (if applicable)					
(1) = (3) **Member State funding**					3.494.155
(2) of which accompanying measures					239.487
(3) of which co-funding a)+b)+c)					3.494.155
a) Public contribution (non EU)					3.211.905
b) Private contribution					282.250
c) Parental contribution					

8 Duration 1 year

9 Involvement of relevant stakeholders

Educational sector	YES	MINISTERIO DE EDUCACIÓN y entidades correspondientes en las Comunidades Autónomas
Public Health	YES	AGENCIA DE SEGURIDAD ALIMENTARIA Y NUTRICIÓN y departamentos responsables de la salud en algunas CCAA
Agriculture	YES	MINISTERIO DE AGRICULTURA, ALIMENTACION Y MEDIO AMBIENTE y Consejerías correspondientes en las CCAA
Civil society	NO	
Private sector	YES	

10 Quantitative indicators

Examples

Participating schools	5.152	56,3%
Participating children	1.161.346	57,3%
Target group	3 to 12	
Specific target group (when relevant and possible)	Not relevant	

Frequency of distributions	Once or twice a week	
Duration of distributions	5 to 24 weeks	
Average consumption per child	Quantity: 2,8Kg	Portions: 19
Delivery time	Morning	Morning break
Delivery system	Trough recognised and authorised distributors	

11 Product

List of products *	**Annex**
Quantities purchased/distributed (indicative)	3.066.740,06 Kg
Per category (fresh, processed) (indicative %)	Fresh: 2.592.791,6Kg (84,54%)
	Processed: 473.949 (15,46%)

Additional criteria (examples)

Child safety (allergic reactions etc.)	Yes
Juice restrictions	According to the law
Additional checks on product safety	Yes
High quality (Integr Pest Managed product, Organic, other (specify)	DOP Peras de Rincón de Soto, Ecological

Availability

Local	Yes
Seasonal	Yes
Community origin	Yes
Organic	Yes
Other	DOP Peras de Rincón de Soto

12 Information

Poster	Yes
Other	Institutional webs, Press, Radio, Leaflets

13 Accompanying measures

Funds allocated (total)		999.685 €
MS funds allocated		10,21 % of total budget
Private funds		
Beneficiaries total		609.614
Recipients total		38,77% of total target group

Measures

Title	Promoting organisation	local regional national
Leaflets, workshops, posters	Public Administration	Regional
Recipe books	Public Administration	Regional
School excursions to the greengrocers	Public Administration	Regional
Organic orchards	Public Administration	Regional
Drawing and photo contests	Public Administration	Regional
School excursions to an agricultural holding	Public Administration	Regional
Educational games	Public Administration	Regional
Website: www.alimentacion.es	Public Administration	National

* Annex- List of products distributed:

Watermelon, tangerine, apple, pear, orange, strawberry, grape, melon, orange juice, apricot, nectarine, plum, kiwi, banana,baby carrots, cherry, cherry tomatoes, mushrooms, peas, beans, cauliflower, lettuce, clementine, grape
